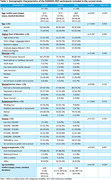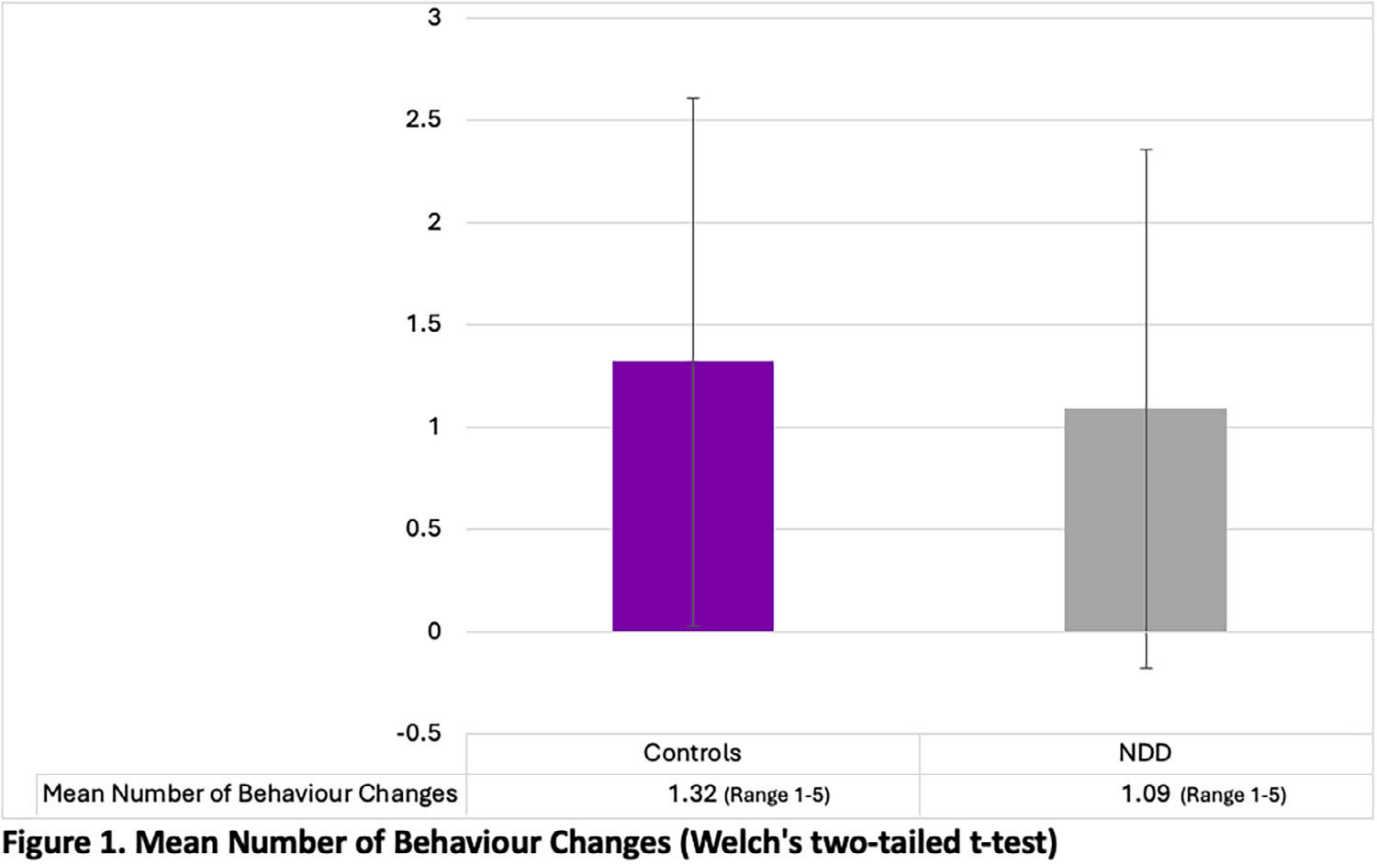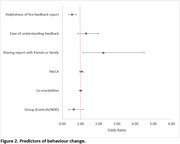# Health Behaviour Changes driven by Personalized Feedback Reports from wearables data: A HANDDS‐ONT Study

**DOI:** 10.1002/alz70860_105589

**Published:** 2025-12-23

**Authors:** Ivan Culum, Emily Narayan, Elizabeth F. Godkin, Kit B. Beyer, Richard H. Swartz, Douglas P. Munoz, Sandra E. Black, Mario Masellis, Anthony E. Lang, Vanessa Thai, Desmond O. Oklikah, William E. McIlroy, Karen Van Ooteghem, Angela C. Roberts, Ontario Neurodegenerative Disease Research Initiative

**Affiliations:** ^1^ Canadian Centre for Activity and Aging, London, ON, Canada; ^2^ Western University, London, ON, Canada; ^3^ University of Waterloo, Waterloo, ON, Canada; ^4^ ICES, Toronto, ON, Canada; ^5^ Sunnybrook Research Institute, Toronto, ON, Canada; ^6^ University of Toronto, Toronto, ON, Canada; ^7^ Queen's University, Kingston, ON, Canada; ^8^ University Health Network, Toronto, ON, Canada

## Abstract

**Background:**

Wearable technologies combined with health feedback can influence health behaviours in older adults and individuals with neurodegenerative diseases (NDD). Despite their potential, limited research explores how health feedback from wearables affects behaviour change in these populations. This study used data from the Health in Aging, Neurodegenerative Diseases and Dementias in Ontario (HANDDS‐ONT) study to examine behaviour changes following the delivery of personalized health feedback reports. Reports were co‐designed with input from clinicians, community stakeholders, and individuals with NDD (Van Ooteghem et al., 2023).

**Method:**

Participants wore wearable devices for 7–10 days, collecting data on physical activity, sedentary behaviour, and sleep. Personalized feedback reports were generated and reviewed in a guided session. After four weeks, participants completed an online survey and interview with a research coordinator assessing feedback utility and behaviour changes. This prospective observational study included participants with at least four days of device wear and survey/interview data (*n* = 203; 98 controls, 105 NDD) (Table 1). Statistical analyses examined group differences (NDD/controls) and predictors of behaviour change, complemented by a thematic analysis of open‐ended survey responses.

**Results:**

No significant group differences were observed in the proportion of participants (Control = 64.3%; NDD = 52.4%) reporting at least one behaviour change (*p* = 0.086, *η* ^2^ = 0.12) or in the number of changes reported (t(200.31) = 1.283, *p* = 0.201, d = 0.180) (Figure 1). A key predictor of behaviour change included sharing the report with family/friends (OR = 2.258, *p* = 0.021). Participants who rated the report as less helpful were less likely to change behaviour (OR = ‐0.527, *p* = 0.001) (Figure 2). Thematic analysis identified subthemes of contemplating change, seeking health information, and social support in behaviour change.

**Conclusion:**

Personalized health feedback reports can encourage behaviour change in individuals with NDD. Perceived helpfulness and social sharing play critical roles. Findings highlight the potential of wearable technologies and tailored feedback in promoting self‐management and health optimization. Future research should explore long‐term behaviour change and the factors influencing sustainability over time.